# Refined pancreatobiliary UroVysion criteria and an approach for further optimization

**DOI:** 10.1002/cam4.4043

**Published:** 2021-08-10

**Authors:** Daniel Mettman, Azhar Saeed, Janna Shold, Raquele Laury, Andrew Ly, Irfan Khan, Shivani Golem, Mojtaba Olyaee, Maura O'Neil

**Affiliations:** ^1^ Department of Pathology and Laboratory Medicine University of Kansas Medical Center Kansas City KS USA; ^2^ Department of Internal Medicine University of Kansas Medical Center Kansas City KS USA

**Keywords:** cytogenetics, cytopathology, FISH, pancreatobiliary, stricture

## Abstract

Pancreatobiliary strictures are a common source of false negatives for malignancy detection. UroVysion is more sensitive than any other method but remains underutilized because of conflicting sensitivities and specificities due to a lack of standardized cutoff criteria and confusion in interpreting results in the context of primary sclerosing cholangitis. We set out to determine the sensitivities and specificities of UroVysion, brushing cytology, forceps biopsies, and fine needle aspiration (FNAs) for pancreatobiliary stricture malignancy detection. A retrospective review was performed of all biopsied pancreatobiliary strictures at our institution over 5 years. UroVysion was unquestionably the most sensitive method and all methods were highly specific. Sensitivity was highest while maintaining specificity when a malignant interpretation was limited to cases with 5+ cells with the same polysomic signal pattern and/or loss of one or both 9p21 signals. Only UroVysion detected the metastases and a neuroendocrine tumor. In reviewing and analyzing the signal patterns, we noticed trends according to location and diagnosis. Herein we describe our method for analyzing signal patterns and propose cutoff criteria based upon observations gleaned from such analysis.

## Graphical abstract

Fluorescent in situ hybridization has been the most sensitive method for detecting pancreatobiliary stricture malignancy in biopsy specimens but is underutilized because of confusion from inconsistent and suboptimal criteria. This article presents refined criteria based on the literature, our experience, and a method for signal pattern analysis that we illustrate in this article.

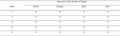

## 1 INTRODUCTION

With up to 20% of surgical resections for suspected biliary malignancy showing benign final pathology, pancreatobiliary stricture malignancy detection is an area where progress needs to be made.^1^ Even with careful clinicopathologic correlation, pancreatobiliary stricture specimens remain a common source of false negatives in general and false positives in primary sclerosing cholangitis (PSC).^1^

UroVysion (U‐FISH), which was developed for urothelial cancer, is the most commonly used ancillary method for biliary strictures but remains relatively underutilized despite repeated reports that it increases sensitivity.^2^, ^3^, ^4^, ^5^, ^6^, ^7^, ^8^, ^9^, ^10^, ^11^, ^12^, ^13^, ^14^, ^15^, ^16^, ^17^, ^18^, ^19^, ^20^, ^21^, ^22^, ^23^, ^24^, ^25^, ^26^, ^27^, ^28^, ^29^ It is a fluorescent in situ hybridization (FISH) assay that uses four probes; 3 pericentromeric probes for chromosomes 3, 7, and 17 as well as a probe for the 9p21 band.^8^ Aneuploidy is a near‐universal event in solid tumors and >66% show whole chromosome alterations.^30^ The chromosomes targeted by U‐FISH contain some of the most commonly implicated solid tumor driver genes and in terms of whole chromosomes aneusomies gain of 7 is the most common gain in solid tumors.^31^ Aneuploidy in malignancies results from caretaker gene alterations that cause DNA repair defects which eventually manifest as chromosomal instability (CSI).^32^ Even though CSI is not an initiating event, it is implicated in cancer progression and the process is nonrandom.^33^ Patterns of aneuploidy are now being associated with different tumors so in addition to being sensitive, detecting aneuploidy can be specific enough in a given case to support one diagnosis over another.^32^

### 1.1 Genetics of malignancies in pancreatobiliary strictures

A summary of the reported cytogenetic abnormalities detectable by U‐FISH for each of the commonly encountered diagnoses in pancreatobiliary strictures can be found in Table 1. For comparison, the most common driver mutations are included along with their corresponding chromosomes. While at least one of the whole chromosome aneusomies involving chromosomes 3, 7, and 17 or 9p21 have been reported in each diagnosis, these findings are more common and significant in some of the diagnoses than others. For most of these tumors, these aneusomies are often late events seen due to CSI, but for gallbladder adenocarcinoma and pancreatic neuroendocrine tumors (pNETs) these are more common and significant, respectively. Carcinomas originating in the gallbladder or cystic duct are genetically heterogeneous and while mutations in many genes have been reported, whole chromosome aneusomies detected by U‐FISH probes are more common than mutations in any of the twenty most associated genes.^34^ In pNETs neoplasia is driven more by aneuploidy than single‐gene mutations.^35^ Lawrence et al. describe two patterns of aneuploidy that correlate with distinct histologic features and clinical outcomes.^35^ Two‐thirds of sporadic pNETs show one of these two patterns which result in the biallelic inactivation of *MEN1* after the loss of heterozygosity in chromosome 11.^35^ Chromosome 3 is the only chromosome represented by U‐FISH that is aneusomic in one of the two patterns.

**TABLE 1:** Genetics table

Tumor	Reported cytogenetic abnormalities (with frequencies where available) detectable by UroVysion	Most common molecular findings (chromosome on which gene sits in parentheses)
Intrahepatic cholangiocarcinoma	Del 9p21^39^	*IDH1* (2), *IDH2* (15), *KRAS* (12), *BRAF* (7), *TP53* (17), *ARID1A* (1), *BAP1* (3), *PBRM1* (3) mutations; *FGFR2* (10) translocations^39^
Extrahepatic cholangiocarcinomas	+3(7%), +7(29%), −3(7%), −7(14%), −9(36%), −17 (36%)^40^	*KRAS* (12), *CDKN2A* (9), *TP53* (17), *SMAD4* (18) mutations; *ERBB2* (17) amplification^39^
Pancreatic ductal adenocarcinoma	+3(22%), +7(19%), +9(11%), +17(6%), −3(17%), −7(3%), −9(25%), −17 (56%)^41^	*KRAS* (12), *TP53* (17), *CDKN2A* (9), *SMAD4* (18), *BRCA1* (17), *BRCA2* (13) mutations^42^
Gallbladder adenocarcinoma	+3(10%), +7(50%), +17(30%), −3(10%), −7(20%), −9(60%), −17 (50%)^43^	*TP53* (17), *KRAS* (12), *CDKN2A* (9) mutations^34^
Ampullary carcinomas	−7, −9, −17^44^	*KRAS* (12), *TP53* (17), *APC* (3) mutations^45^
Acinar carcinoma	3 infrequently lost or gained, +7 > −7, −9 > +9, +17 > −17^46^	*APC* promoter (3) methylation>loss>> mutation^47^
pNET	−3^35^	Biallelic *MEN1* (11) inactivation^35^

Abbreviation: pNET, pancreatic neuroendocrine tumor.

### 1.2 Biliary U‐FISH

Since the first report on the use of U‐FISH in pancreatobiliary strictures, U‐FISH has repeatedly been shown to be more sensitive than cytology with reported sensitivities from 31% to 84% and specificities from 83% to 100%.^12^, ^13^, ^24^ FISH is less specific in patients with PSC and in such cases, serial measurements are important to prevent false positives.^29^, ^36^, ^37^ The main weakness of U‐FISH for pancreatobiliary strictures is the lack of standardized and optimized criteria; this explains the large range of sensitivities and specificities and provides a promising area for improvement in maximizing preoperative malignancy detection.

The most common polysomic threshold is five cells with more than two copies of at least two of the probes, excluding tetrasomy. Some people use four cells as is done in urine.^17^ While the 9p21 probe was initially disregarded, it has become apparent over time that 9p21 can be indicative of malignancy even in the absence of polysomy. This is the main area of confusion as criteria for 9p21 loss have varied tremendously. Proposed criteria have included: loss of one or both signals in five cells, loss of one signal in 10 cells, loss of two signals in 10 cells, loss of one or both signals in 12 cells, loss of both signals in 5%, loss of one signal in 6%, and loss of signals in 20% cells.^5^, ^12^, ^13^, ^17^, ^18^, ^21^ While trisomy 7 was once considered sufficient for malignancy detection by some it has become evident that its presence is essentially noncontributory in malignancy detection.^4^, ^16^, ^21^ It is usually grouped with trisomy 3 and its presence is simply noted or an equivocal interpretation is rendered when either is seen in 10 cells or 10% of cells. ^21^, ^29^ Tetrasomy has been deemed a nonspecific finding and either grouped with trisomy or its presence specifically noted when seen in 10 cells or 10% of cells.^21^, ^29^ In PSC, polysomy can be seen in the absence of malignancy and reverts over half the time, so serial or multifocal positivity and clinical evidence of malignancy are necessary.^9^, ^11^, ^20^ In PSC, positive FISH findings can be seen in dysplasia.^38^ Polysomy can be seen over a year before pathologic or radiologic evidence of malignancy.^11^ For these reasons, it is recommended that FISH be repeated when polysomy is detected in the absence of other evidence of malignancy and close monitoring of repeat polysomic results is recommended despite the length of time elapsed since the initial polysomy result.^11^ Although established as the most clinically relevant ancillary technique, with professional associations such as the Papanicolaou Society of Cytopathology even recommending its routine usage, U‐FISH is rarely used for pancreatobiliary strictures.^2^ This is likely at least in part due to initial reports of low specificity resulting from trisomy 7 criteria being applied as independently sufficient for malignancy detection as well as from a lack of understanding as to how to interpret results in the context of PSC. Rare experiments with different FISH probes have been no more sensitive than U‐FISH when both polysomy and 9p21 criteria are used.^21^ While one might surmise that advances in next‐generation sequencing (NGS) would surely allow for a clinically useful panel of sufficient size to supersede U‐FISH, Dudley et al. reported their NGS panel containing nearly every driver gene from Table 1 to be only comparable to U‐FISH when 9p21 criteria were not used.^25^ As an in situ technique, and in contrast to NGS, FISH allows visual confirmation that the mutation is present in and limited to the morphologically atypical cells. In comparison to NGS, FISH is far more sensitive, requiring fewer atypical cells and a lower tumor fraction.^21^ While resections are performed for cases that are benign but thought to be malignant, this is usually the result of resection based solely upon radiologic findings when a biopsy cannot be obtained or when biopsies have been repeatedly negative despite concerning clinical and radiologic findings. The main issue is insensitivity. Pancreatobiliary strictures and malignancies are notorious for yielding paucicellular lesional samples because of the effects of fibrosis and desmoplasia. For this reason, and because of the particularly low tumor fraction with the often abundant non‐lesional gastrointestinal epithelial contamination that inevitably results because of the difficulty in sampling these lesions, FISH may provide unique promise in this area.

In this paper, we report on the 5‐year experience of a large academic medical center in using cytology, forceps biopsies, and U‐FISH to diagnose pancreatobiliary strictures. We provide insights from signal pattern analysis and criteria recommendations that in our experience have increased sensitivity while preserving specificity.

## 2 MATERIALS AND METHODS

### 2.1 Patient population

At the approval of the institutional review board, the cytogenetics database was queried to identify all pancreatobiliary brushing U‐FISH specimens at our institution from October 2014 to November 2019. From this list, a patient list was generated and no patients or specimens were excluded.

### 2.2 Specimen collection

All strictures were biopsied with some combinations of brushing ± forceps biopsy and/or FNA. For each case, one brushing was placed in CytoLyt (Hologic Inc.) for the creation of a ThinPrep (Hologic Inc.) and another was placed in saline for UroVysion (Abbott Molecular Inc.). Forceps biopsies were collected in formalin. FNAs were collected in CytoLyt for the creation of ThinPreps.

### 2.3 FISH interpretation

A cytogenetic technologist screened the slides and recorded signal patterns on a score sheet before a cytogeneticist reviewed the cases and produced final reports. Specimens were considered positive if criteria were met for polysomy or 9p21 loss. Polysomy was defined as three or more signals for two or more probes in five or more cells unless there were exactly four signals for each probe. Loss of 9p21 was defined as loss of one or two 9p21 signals in five or more cells. Specimens were considered equivocal if they had 10 or more cells with tetrasomy or trisomy. All other specimens were considered negative.

### 2.4 Signal pattern analysis

**FIGURE 1:**
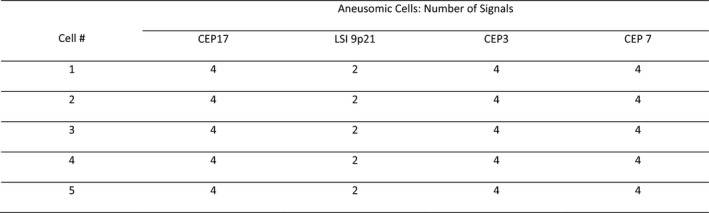
This is an abridged representation of an actual score sheet from one of the pancreatic ductal adenocarcinoma (PDAC) cases. The signal patterns could mistakenly be attributed to independent gains of 3, 7, and 17. This pattern is instead the result of hemizygous loss of 9p21 followed by whole‐genome doubling. As shown in Table 1, in PDACs loss of 9 is more common than the gain of 3, 7, or 17

**FIGURE 2:**
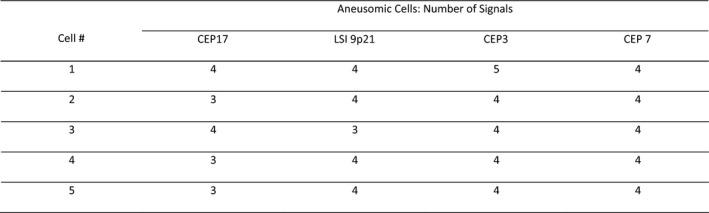
This is an abridged representation of an actual score sheet from one of the pancreatic ductal adenocarcinoma (PDAC) cases. The signal patterns can be seen to be due to cytogenetic instability in a tetrasomic cell population instead of from gains in a diploid population. Based on the frequencies with which each aneusomy is seen in PDAC (listed in Table 1) as compared to the frequency of whole‐genome doubling (WGD) in solid tumors, it is far more likely that this many chromosomes with exactly four signals would be due to WGD than independent gains. Furthermore, when a number other than four signals is present, the number is either one less or one more than four. When three signals are seen the possibilities could be that the cell gained a copy of that chromosome or that there were four copies and one was lost. When three of five cells contain three signals for a given probe and the other cells contain four signals for said probe it is usually more likely that copies were lost, especially when the probe in question is CEP17 and the lesion is a PDAC. Loss of 17, loss of 9p21, and gain of 3 are the three most common whole chromosomal aneusomies affecting UFISH probes and occur in a 3:1:1 as illustrated by the ideogram from Kowalski et al. When the observed signal patterns could not be explained by the expected aneusomic frequencies it was denoted in the inferred cytogenetic sequence by RLAG (random losses and gains)

**FIGURE 3:**
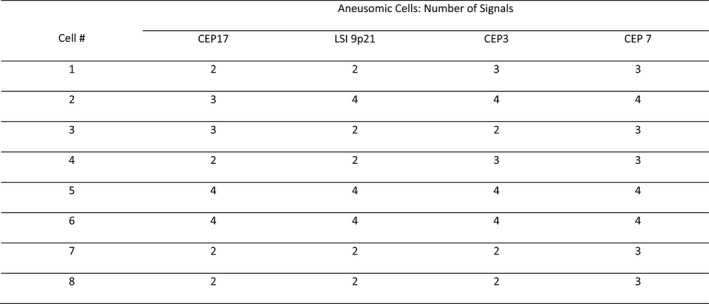
This is an abridged representation of an actual score sheet from a benign case. If one were using polysomy in four cells as the threshold for malignancy this would be interpreted as positive since Cells 1–4 would be called polysomic. Cell 2 may simply represent a tetrasomic cell that randomly lost one CEP17 signal since there are other tetrasomic cells and chromosome 17 is frequently lost. In cases with multiple tetrasomic cells, it is common to see one or more that have lost a copy of CEP17 or another probe. This case illustrates why we believe that positivity should require five instead of four polysomic cells, that the polysomic cells have the same signal pattern, and that cases with tetrasomic or near‐tetrasomic cells be interpreted with caution

**FIGURE 4:**
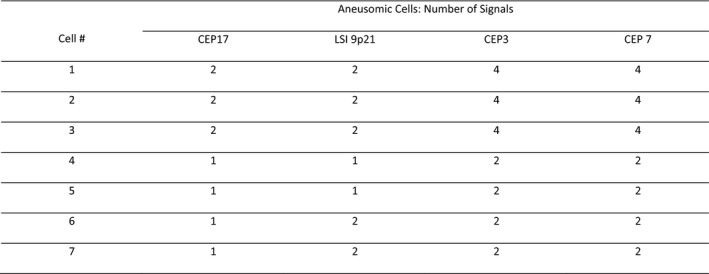
This is an abridged representation of an actual score sheet from a pancreatic ductal adenocarcinoma (PDAC) case. This example demonstrates how one can deduce the order of events from the different populations of cells. Looking at Cell 1 one may wonder whether 3 or 7 was gained first and may not consider that 17 and 9p21 were lost. Cell 4 having only one CEP17 signal and one 9p21 signal reveals that at least some cells lost these signals at some point. After noticing this, it becomes evident that Cells 1–3 represent cells such as Cell 4 that have undergone whole‐genome doubling (WGD). This tells you that the losses of both 17 and 9p21 occurred prior to WGD. The presence of cells such as Cells 6 and 7 combined with the absence of cells showing loss of only 9p21 suggests that loss of 17 preceded loss of 9p21. When the signal patterns of the different cells did not allow for the determination of one event preceding or following another it was denoted in the inferred cytogenetic sequence by OCBD (order cannot be determined)

Score sheets were reviewed to ensure the proper interpretation of signal patterns and to generate an inferred cytogenetic sequence for each case. The cytogenetic sequence responsible for the observed signal patterns in a case was inferred from the consideration of the reported relative frequencies of each aneusomy detectable by U‐FISH for a particular diagnosis as well as how these would most likely result in the observed signals. See Table 1 for reported frequencies of U‐FISH‐detectable aneusomies and Figures 1, 2, 3, 4 for a stepwise approach and examples that clarify the signal pattern analysis process.

### 2.5 Morphologic interpretation

Pancreatobiliary cytology specimens that showed atypical cells of quantitative or qualitative insufficiency for a malignant interpretation were diagnosed as atypical with a qualifier understood by our clinicians to indicate the pathologist's level of concern. The qualifiers in order of increasing suspicion include “cannot exclude malignancy,” “suspicious for malignancy,” “favor malignancy,” and “consistent with malignancy.”

### 2.6 Other data collection

The medical record for each patient was comprehensively reviewed. To call a case benign there had to be a lack of evidence of malignancy on follow‐up imaging at least 12 months after the brushing collection as well as a known benign candidate etiology and complete stricture resolution without the resolution being due to resection, chemotherapy, or radiation. To call a case malignant there had to be histologic evidence or both compelling radiologic evidence of metastasis and the absence of another candidate lesion for the site of origin.

## 3 RESULTS

The study included 181 encounters and 154 patients. There were 126 encounters in 113 patients whose strictures were ultimately determined to be of benign etiology. There were 55 encounters in 41 patients whose strictures were ultimately determined to be of malignant etiology. In total, there were 52 encounters in 39 patients with PSC. Other than the one false positive, PSC cases did not show different FISH signal patterns from other benign and malignant cases. Of these, 43 of the encounters occurred in the 34 patients whose strictures were ultimately determined to be of benign etiology. Overall, there were 20 people with multiple encounters resulting in 27 repeat encounters. Repeat encounters for malignant lesions (*n* = 11) were excluded from analysis for calculating sensitivities of individual methods. Of the 55 malignant lesions, 14 had only negative encounters. If an encounter with multiple specimen types consists entirely of negative results for a lesion that is known to be malignant, it is more likely that the multiple concurrent specimens are all negative as a result of failing to sample the lesion than as a result of the insensitivity of the techniques. Sensitivities calculated by excluding such negative encounters can highlight and reduce the effect of sampling. This may more accurately represent the sensitivity of the technique and allow for a more meaningful comparison between results from specimens obtained by different endoscopists and from different lesions.

Brushing cytology and U‐FISH were attempted in all cases, but in 12 cases (nine benign, three malignant) the brush for U‐FISH contained insufficient cells and in three cases (two benign, one malignant) the cytology brush resulted in an acellular liquid‐based preparation. It was more common for the FISH brush to contain insufficient cells but it was always collected after the cytology brush. This and there being no cases in which both were insufficient indicate that such cases are more likely the result of the collection than insensitivity of the analytical method. For this reason, insufficient results were excluded from statistical analysis instead of being considered as true or false negatives.

Table 2 shows the sensitivities and specificities of each method at different cutoffs. FISH was by far the most sensitive method. All methods were of high specificity. The one false‐positive FISH was in a case of PSC and showed an increase in all probe signals in seven cells, increases in all probe signals with no 9p21 signals in three cells, increased signals for all but the 9p21 probe in eight cells, increased CEP3 and CEP7 signals in four cells, increased CEP7 and CEP 17 signals with no 9p21 signals in one cell, an increase in CEP7 and only one 9p21 signal in one cell, and an increase in the CEP7 signal in one cell. There were ten cases in which only U‐FISH was positive. This included four pancreatic ductal adenocarcinomas (PDACs), two distal cholangiocarcinomas (DCs), one intrahepatic cholangiocarcinoma (IHC), both metastases and one of the three pNETs. Only brushing cytology was positive for one IHC and one PDAC. There were no cases where only forceps biopsy or FNA was positive.

**TABLE 2:** Sensitivities and specificities of individual modalities

Interpretations considered positive	Sensitivity (%)	Specificity (%)
Brushing cytology (*n* = 178)		
Adenocarcinoma only	24	100
Suspicious, favor, consistent with, and adenocarcinoma	46	100
Suspicious, favor, consistent with, and adenocarcinoma excluding negative encounters^a^	63	100
Forceps biopsy (*n* = 68)		
Adenocarcinoma only	24	100
Suspicious, favor, consistent with, and adenocarcinoma	36	100
Suspicious, favor, consistent with, and adenocarcinoma excluding negative encounters	47	100
FNA (*n* = 39)		
Adenocarcinoma only	32	100
Suspicious, favor, consistent with, and adenocarcinoma	39	100
Suspicious, favor, consistent with, and adenocarcinoma excluding negative encounters	60	100

Brushing FISH (*n* = 169)	Sensitivity (%)	Specificity (%)
Criteria for positive interpretation		
Polysomy	58	99
Polysomy or loss of 9p21	71	99
Polysomy or loss of 9p21 excluding negative encounters	95	99

^a^ Negative encounters consist of encounters with multiple specimens all with negative results for a lesion that is independently confirmed to have been malignant. See the main text for an explanation as to why this is a meaningful value to report.

Table 3 contains the diagnoses, FISH interpretations, abnormal signal patterns, and inferred cytogenetic sequences for each malignant case. From the ERCP reports, the precise location of the stricture was determined and cholangiocarcinomas were divided into intrahepatic, proximal extrahepatic, and distal extrahepatic types. If the stricture was centered proximal to the common hepatic duct origin, it was classified as IHC. If the stricture was centered in the common hepatic duct it was classified as extrahepatic proximal cholangiocarcinoma. If the stricture was centered distal to the cystic duct origin it was classified as DC.

**TABLE 3:** Malignant case FISH findings

Eventual diagnosis	FISH interpretation	Abnormal cell signal patterns			Inferred cytogenetic sequence	
IHC^a^	Positive	•P3/7/17^b^ L9(2)^c^ (12 cells)	•P3/7/17 L9(1) (2 cells)		•L9(1) then WGD^d^	
		•P7/17 L9(2) (1 cell)			•L9(2) then WGD	
	Positive	•P3/7/9/17 (6 cells)	•TE^f^ (6 cells)		•TR9 then WGD	
		•TR9^e^ (2 cells)				
	Positive	•P3/7/9/17 (7 cells)			•G17^g^ then G7	
		•TR7 (10 cells)			•TR7	
	Positive	•TR7 L9(2) (25 cells)			•TR7 and L9(2) (OCBD)^h^	
	Positive	• TR7 L9(1) (25 cells)			•TR7 and L9(1) (OCBD)	
	Positive	•TR3 L9(2) (28 cells)			•TR3 then L9(2)	
		•TR3 (8 cells)				
	Positive	•L9(1) (10 cells/10% of total)			•L9(1)	
	Positive	•L9(2) (6 cells)			•L9(2)	
	Negative	•P3/7 (1 cell)	•TR3 (1 cell)		N/A^i^	
	Negative	•P3/7/17 L9(2) (1 cell)	•TE (1 cell)		N/A	
IHC (PSC)^j^	Positive	•G3 L9(2) (24 cells)	•TR3 (1 cell)		•TR3 then L9(2)	
	Negative	Insufficient cells for U‐FISH^k^			N/A	
EHPC^l^	Positive	•P3/7/17 L9(2) (25 cells)			•TR7 and L9(2) (OCBD) then WGD	
	Positive	•P3/7/9/17 (5 cells)	•P7/9/17 (3 cells)	•TR3 (1 cell)	•WGD then RLAG^m^	
		•P3/7/9 (1 cell)		•TR7 (1 cell)	•TR3	
		•P3/9/17 (2 cells)	•TE (5 cells)		•TR7	
	Positive	•P3/7/9/17 (17 cells)			•G3 then G17 then WGD	
		•P3/7/17 (8 cells)			•G3 then G17 then G7 then WGD	
	Positive	•P3/7/9/17 (7 cells)	•P3/7/17 (6 cells)	•P3/17 (1 cell)	•L9(1) then WGD	
		•P3/7/9 (3 cells)		•TE (17 cells)	•WGD then L9(1)	
			•P7/9/17 (1 cell)			
	Negative	Insufficient cells for U‐FISH			N/A	
EHPC (PSC)	Positive	•P3/7/17 L9(2) (1 cell)	•P3/7/9/17 (21 cells)	•P3/7 (3 cells)	•L9(1) then WGD	
	Positive	•P3/7/17 (25 cells)			•L9(2) then WGD	
DC^n^ (involving proximal CBD and distal CHD)	Positive	•P3/7/9/17 (14 cells)	•P7/9/17 (1 cell)	•TR7 (19 cells)	•WGD then RLAG	•TR7
			•TE (22 cells)	•TR3 (1 cell)		•TR3
		•P3/7/9 (1 cell)				
	Negative	Insufficient cells for U‐FISH			N/A	
DC (involving proximal CBD and distal CHD) (PSC)	Positive	•P3/7/9/17 (12 cells)			•TR7 then G3	
		•TR7 (23 cells)			•TR7 then G9	
					•TR7 then WGD	
IPNB (proximal CBD)	Positive	•L9(1) L17(1) (109 cells)	•P3/7 (17 cells)		•L9(1) then L17(1) then WGD	
		•P3/7/9 (1 cell)	•G3 (3 cells)			
DC (middle CBD)	Positive	•P3/7/9 (1 cell)	•P3/7/17 (18 cells)	•P3/7 (6 cells)	•L9(1) then WGD then L17(1) G7	
	Positive	•P3/7/17 (22 cells)	•P3/7 (1 cell)	•P3/17 (1 cell)	•L9(1) then WGD	
DC (distal CBD)	Positive	•P3/7/9/17 (11 cells)			•WGD then RLAG	
	Negative	NASP^o^			N/A	
Mixed acinar‐ductal pancreatic carcinoma	Positive	•P3/7/17 L9(2) (2 cells)	•P7/17 L9(2) (10 cells)	•TR17 L9(2) (15 cells)	•L9(2) then G17 then G7	
					•L9(2) then G17 then WGD	
PDAC^p^	Positive	•P3/7/17 L9(2) (12 cells)	•TR7 (11 cells)		•L9(2) then WGD	
					•TR7	
	Positive	•G7 L9(2) (6 cells)	•P3/7/9 (6 cells)	•P3/7 (1 cell)	•TR7 then L9(2)	
		•P3/7/9/17 (2 cells)	•P7/9 (4 cells)		•TR7 then G9 then G3	
	Positive	•P/3/7/17 L9(2) (25 cells)			•L9(2) then WGD	
	Positive	•P3/7/9/17 (9 cells)	•P7/9/17 (1 cell)	•TE (1 cell)	•WGD	
		•P3/7/17 (2 cells)	•P7/17 (1 cell)			
	Positive	•P3/7/9/17 (5 cells)	•P7/9/17 (2 cells)	•TE (4 cells)	•L9(1) then WGD	
		•P3/7/17 (9 cells)				
	Positive	•P3/7/17 (12 cells)	•TE (2 cells)		•L9(1) then WGD	
	Positive	•P3/7/17 L9(2) (8 cells)	•TR7 (1 cell)		•L9(2) then WGD	
					•TR3	
		•TR3 (2 cells)			•TR7	
	Positive	•P3/7/9/17 (16 cells)	•P3/7/17 (1 cell)		•WGD	
		•P3/7/9 (3 cells)	•P7/9 (1 cell)			
	Positive	•L9(2) (75 cells)	•TR7 (1 cell)		•L9(2)	
		•TR3 L9(2) (1 cell)	•G3 (1 cell)		•TR3	
					•TR7	
	Negative	•P3/7/9/17 (1 cell)	•P3/7/17 (1 cell)	•P3/7 (1 cell)	N/A	
	Negative	•TR7 (10 cells)	•TR7 L17 (2 cells)		N/A	
	Negative	•TR7 (8 cells)			N/A	
	Negative	•P3/7 (2 cells)			N/A	
	Negative	•TE (1 cell)			N/A	
	Negative	•L17(1) (13 cells)			N/A	
	Negative	NASP			N/A	
	Negative	NASP			N/A	
	Negative	NASP			N/A	
Ampullary carcinoma	Positive	•P7/9/17 (13 cells)	•G7 (2 cells)		•G7 then G9 G17 (OCBD)	
Gallbladder adenocarcinoma	Positive	•P3/7/17 L9(2) (25 cells)	•L9(2) then WGD			
	Positive	•P3/17 (21 cells)	•P3/7/9/17 (3 cells)	•P3/9/17 (1 cell)	•G3 then G17 then WGD	
	Negative	•L9(1) G17 (1 cell)	•TE (1 cell)		N/A	
pNET^q^	Negative	•Trisomy 7 (11 cells)			N/A	
	Positive	•P3/17 L9(1) (1 cell)	•P3/7/17 (1 cell)	•L9(1) L3(1) (5 cells)	N/A	
		•P3/7 (1 cell)	•TE (4 cells)			
	Negative	•TR3 (1 cell)	•TR7 (1 cell)	•L17(1) (3 cells)	N/A	
Metastatic urothelial carcinoma	Positive	•P3/7/9/17 (19 cells)	•P3/7/9 (4 cells)	•P3/7/17 (2 cells)	•WGD	
Metastatic colonic adenocarcinoma	Positive	•P3/7/9/17 (23 cells)	•P3/7/9 (2 cells)		•WGD	

Note

Each row contains the FISH findings for a single malignant case. Each bullet point represents a population of cells.

^a^ IHC, intrahepatic cholangiocarcinoma (stricture centered proximal to common hepatic duct origin).

^b^ P followed by numbers separated by slashes denotes a polysomic cell and which chromosomes contained more than two signals.

^c^ L followed by a number denotes a signal loss in the chromosome represented by the number with the number of lost signals for that chromosome indicated in parentheses.

^d^ WGD, whole‐genome doubling.

^e^ TR, trisomy.

^f^ TE, tetrasomy.

^g^ G, gain in a single chromosome, the signal gained.

^h^ OBCD, order cannot be determined, used when multiple changes (e.g., a gain and a loss) occurred but it is impossible from the observed signal patterns to deduce in what order they occurred.

^i^ N/A, not applicable.

^j^ PSC, primary sclerosing cholangitis.

^k^ U‐FISH, UroVysion.

^l^ EHPC, extrahepatic proximal cholangiocarcinoma (stricture centered in common hepatic duct).

^m^ RLAG, random losses and gains, used when there is obviously no pattern to the observed gains and losses.

^n^ DC, distal cholangiocarcinoma (stricture centered distal to cystic duct origin).

^o^ NASP, no abnormal signal patterns.

^p^ PDAC, pancreatic ductal adenocarcinoma.

^q^ pNET, pancreatic neuroendocrine tumor.

## 4 DISCUSSION

### 4.1 The importance of signal pattern analysis

The pancreatobiliary U‐FISH literature describes polysomy as if it usually results from multiple independent gains when in actuality most polysomic cells in the context of pancreatobiliary malignancy are the result of whole‐genome doubling (WGD). Unlike benign cells, which are only tetrasomic after DNA replication during the S phase and until cytokinesis, malignant cells are often tetraploid or near‐tetraploid outside this window due to aberrancies in DNA replication or cell division. Tetraploidy can result in multipolar spindle formation and cytogenetic instability. The role of WGD in bringing about polysomy has not been emphasized and polysomy has been touted as the single most important U‐FISH finding, while tetrasomy has been assessed as nonspecific and something that must be distinguished from polysomy so as to preserve the specificity of polysomy. Polysomy is, indeed, more specific for malignancy than tetrasomy, but it is important to recognize tetrasomy, WGD, and the role they play in generating polysomy. Analysis of signal patterns with tetrasomy and WGD in mind elucidates the sequence of cytogenetic changes that resulted in the detected polysomy. Figure 3 illustrates the potential consequences of simply looking for polysomic cells without considering the cytogenetic mechanisms responsible for the polysomic cells.

In most cases, a review of the U‐FISH signal patterns from a polysomic case reveals that the polysomic cells arose out of tetraploid cells or otherwise abnormal cells that underwent WGD. Examples and further explanations of this can be found in Figures 1, 2, and 4. Of the 37 malignant cases detected by U‐FISH, sequential gains preceded WGD in only five cases (see Inferred Genetic Sequence column of Table 3). Moreover, 9p21 loss preceded WGD in 62% (23/37) of cases. This explains why optimization of 9p21 criteria is key to improving sensitivity, especially sensitivity for earlier stage cases that are more likely to be amenable to resection and in which FNA may be contraindicated.

### 4.2 Proposed criteria based on signal pattern analysis

#### 4.2.1 Same polysomic signal pattern in five cells = positive for malignancy

Our data support a criterion requiring five polysomic cells that show the same signal pattern. Fewer cells may be too nonspecific as we had two negative cases with three polysomic cells. See Figure 3 for an example that illustrates the importance of requiring the same signal pattern. Requiring five cells of the same type seems reasonably restrictive as all of our positive malignant cases and none of our benign cases except the one false‐positive case met this criterion. Trying to adapt the criteria to exclude the false‐positive case would result in unacceptably low sensitivity and would be unnecessary since the false positive was in a person with PSC in whom one should know not to overvalue a single positive polysomic result.

#### 4.2.2 Loss of one or two 9p21 signals in even five cells = positive for malignancy

Our data support the 9p21 criterion for malignancy proposed by Kubiliun et al. of five cells with either a homozygous or hemizygous loss.^12^ While there are occasional benign cases that show the rare cell with 9p21 signal loss, the most we saw in a benign case was three cells except for our one known false positive. We had one IHC and one PDAC each with only six cells showing homozygous 9p21 signal loss as well as a pNET with hemizygous 9p21 signal loss in only five cells, so requiring more than five cells would likely result in a significantly reduced sensitivity. Others have proposed 5% but when few cells are present this could result in overcalling malignancy.

#### 4.2.3 Tetrasomy in 10% = equivocal

While tetrasomic cells are encountered in benign specimens, they are usually few in number. Barr Fritcher et al. report that 40% of cases showing 10 or more tetrasomic cells are malignant.^15^ The most we saw in a benign case was seven tetrasomic cells in a case where 73 cells were counted. For this reason, we propose that any cell containing 10% tetrasomic cells be noted.

#### 4.2.4 Trisomy 7 alone = negative

Although it is now generally accepted that cases with trisomy 7 are more likely to be benign than malignant, there have been people who considered the presence of trisomy 7 as sufficient for malignancy detection.^4^, ^12^, ^14^, ^16^ As with other criteria, different thresholds have been proposed to achieve higher specificity. In our cohort, there were benign cases in patients with and without PSC that each had trisomy 7 in more than 25 cells and more than 30% of the cells. We had benign cases where trisomy 7 was no longer apparent a month later and others where it persisted for more than 3 years, so even persistence is not diagnostic of malignancy.

For these reasons, we do not think there is any threshold at which trisomy 7 alone should be considered positive for malignancy detection. We recommend against an equivocal interpretation because of the frequency with which trisomy 7 is seen and the devaluation of an equivocal interpretation if used so commonly and in so many cases that end up benign.

#### 4.2.5 Trisomy 3 in 10+ cells = equivocal

Since U‐FISH has been used on pancreatobiliary strictures trisomy 3 has been regarded similarly to trisomy 7 with many reports even lumping the two together without specifying which is present. But while trisomy 7 is seen very frequently in benign pancreatobiliary strictures, trisomy 3 is rarely seen in benign pancreatobiliary strictures.

Only seven cases of pancreatobiliary strictures with trisomy 3 have been reported and only one was malignant.^4^, ^12^, ^15^, ^24^ Only Kubiliun et al. reported the number of cells with trisomy 3 for each of their cases.^12^ It was specified that 2 of their benign cases had fewer than 10 cells with trisomy 3, while a third case had 12 cells with trisomy 3.^12^ The malignant case, reported by Chaiteerakij et al., was a PDAC in which the cells with trisomy 3 were also homozygous for loss of the 9p21 locus.^24^

Our data significantly add to what has been reported and show trisomy 3 to be both common in malignant cases as well as more common in malignant than benign cases. Trisomy 3 cells were seen in one benign case, one negative specimen targeting a subsequently confirmed malignancy, and five malignant specimens. Two DCs and one PDAC each showed a single cell with trisomy 3. All three cases contained tetraploid populations and the complex variety of signal patterns typical of such cases, so in these cases, the trisomy is likely of little significance and represents a random event. In one IHC 19 cells showed trisomy 3, 18 of which also showed homozygous 9p21 loss. In another IHC 36 cells showed trisomy 3, 28 of which also showed homozygous 9p21 loss.

While we have recommended refraining from an equivocal interpretation for trisomy 7, our data support attention to trisomy 3, especially when seen in an intrahepatic lesion and even more so when seen in greater than 10 cells. The one reported benign case that had more than 10 cells with trisomy 3 was a PDAC. Other than the cases reported by Kubiliun et al., all previously reported benign cases with trisomy 3 were from studies that did not consider 9p21 probe status in assessing for malignancy detection. Malignancy was only detected preoperatively by the 9p21 probe in both of our IHCs with trisomy 3 which raises the possibility that previous cases assumed to be benign without consideration of 9p21 status were actually malignant.

#### 4.2.6 Trisomy 9 in five cells or trisomy 17 in five cells = equivocal

Trisomy 9p21 and trisomy 17 are seen far less frequently than trisomy 7 and trisomy 3. Our cohort contained one case with trisomy 9p21 and one with trisomy 17. To our knowledge, this is the first report of either of these findings in the literature on FISH for the detection of pancreatobiliary malignancy. Trisomy 9p21 was seen in a case of IHC. In the polysomic cells, there were more signals for 9p21 than for any of the other probes and there were most often four signals for the other probes. There was a separate population of cells showing only trisomy 9p21 and another population of tetrasomic cells. This constellation of findings suggests trisomy 9p21 preceded the polysomy and that the polysomic cells simply represent cells with 9p21 trisomy that underwent WGD.

### 4.3 Other insights from signal pattern analysis

#### 4.3.1 Signal patterns vary by location and diagnosis

Discrepancies between previously reported sensitivities and specificities may be explained by our finding that signal patterns vary by location and diagnosis. WGD occurs later in IHC than in extrahepatic cholangiocarcinoma (EHC) or PDAC. At the time of the first positive U‐FISH result, WGD was seen in 33% of IHC (3/9), 100% of EHC (12/12), and 78% of PDAC (7/9). Because WGD occurs late in IHC, polysomy criteria are often not positive at the time of brushing. Polysomy criteria were not met in 67% (6/9) of detected IHCs and said cases were only detected by 9p21 criteria.

Hemizygous 9p21 signal loss is far less reported than homozygous loss and it has even been suggested that hemizygous loss is specific to low‐grade dysplasia.^37^ Our data show that not only is hemizygous 9p21 loss seen at a frequency similar to that of homozygous loss in pancreatobiliary malignancy, but that this is underappreciated without the consideration of WGD and that there may be a location‐dependent pattern to 9p21 loss. In our cohort, homozygous and hemizygous 9p21 losses were seen with near equal frequencies in IHCs, perihepatic EHCs, and PDACs. However, all three DCs with 9p21 loss showed hemizygous loss.

#### 4.3.2 Normal or decreased number of CEP3 signals in extrahepatic lesion may indicate a less common diagnosis

While most malignant pancreatobiliary strictures are the result of PDAC or cholangiocarcinoma, several other diagnoses can present as pancreatobiliary strictures. We had several noteworthy cases that are all relatively rare in pancreatobiliary strictures and otherwise unique in that they are the only positive cases in our cohort outside the liver that showed a normal number of CEP3 signals. They included an ampullary carcinoma with intestinal differentiation, a mixed ductal‐acinar pancreatic carcinoma (confirmed via outside expert consultation), an intraductal papillary neoplasm of the bile duct (IPNB), and a pNET. This could be useful in alerting one to consider a less common diagnosis which is particularly helpful in this area of oncology where immunohistochemistry is ineffective in determining tissue of origin and malignant cases are usually diagnosed as adenocarcinoma with clinicians expected to determine the specific diagnosis based on the clinical presentation. While clinical correlation is reasonable for differentiating PDACs and cholangiocarcinomas, it is insufficient for distinguishing the less common entities which are often misdiagnosed until resection.

To summarize, polysomy in pancreatobiliary malignancy most often results from WGD as opposed to multiple independent gains. In the majority of cases, 9p21 loss precedes WGD, but WGD in the absence of 9p21 loss and WGD in the absence of sequential independent gains are each responsible for polysomy in nearly onefifth of such cases. Signal patterns vary by location and diagnosis. The CEP3 signal appears to be affected in all primary tumors encountered in pancreatobiliary strictures except IHC, intestinal ampullary carcinoma, pancreatic acinar carcinoma, and the in‐situ component of IPNB. Awareness of these principles is important because it informs how one interprets the sequence of cytogenetic events and this sequence may provide diagnostic information that is not apparent without signal analysis.

In conclusion, we provide new criteria that could increase the sensitivity while maintaining the specificity of U‐FISH for malignancy detection in pancreatobiliary strictures. Our report is particularly contributory for its level of detail on stricture location, FISH signal pattern, and signal pattern analysis. Subsequent studies on specific signal patterns and the changes responsible for them may allow for even more refined criteria that could improve upon the already superior sensitivity and near‐perfect specificity of U‐FISH in this area where current methods are unable to accurately detect malignancy preoperatively in a remarkably high proportion of cases.

## 5 ANALYZING SIGNAL PATTERNS

*Step 1*. Identify the cell(s) with the greatest number of signals for a single probe.

*Step 2*. Determine the most probable sequence of events that could result in a cell with this signal pattern.

- If there is no cell with at least four signals for one or more probes, then the number of gains and losses is usually few and can be explained simply by the relative expected frequency of each aneusomy for each probe for the tumor type.
- If the number of signals for any of the probes is four or more in any of the cells, one should first determine if it is most likely that such cells arose from tetrasomic cells, aneusomic cells that underwent WGD, or from a series of gains and losses in diploid cells. This requires and is facilitated by consideration of the other observed signal patterns.

*Step 3*. Repeat steps 1 and 2 until all observed signal patterns have been accounted for.

## 6 ETHICAL APPROVAL STATEMENT

This project was approved by the Institutional Review Board of the Human Research Protection Program at the University of Kansas Medical Center. It conforms with the US Federal Policy for the Protection of Human Subjects.

## CONFLICT OF INTEREST

None of the authors have any conflicts of interest to report.

## AUTHOR CONTRIBUTION

Daniel Mettman, Shivani Golem, Mojtaba Olyaee, and Maura O'Neil contributed to the conception, design, data acquisition, data analysis, manuscript drafting, and manuscript revision; Azhar Saeed, Janna Shold, Raquele Laury, Andrew Ly, and Irfan Khan contributed to data acquisition, data analysis, manuscript drafting, and manuscript revision.

## Data Availability

The data that support the findings of this study are available from the corresponding author upon reasonable request.
